# Publisher Correction: Power generator driven by Maxwell’s demon

**DOI:** 10.1038/s41467-023-37321-2

**Published:** 2023-03-27

**Authors:** Kensaku Chida, Samarth Desai, Katsuhiko Nishiguchi, Akira Fujiwara

**Affiliations:** NTT Basic Research Laboratories, NTT Corporation, 3-1 Morinosato Wakamiya, Atsugi, Kanagawa 243-0198 Japan

Correction to: *Nature Communications* 10.1038/ncomms15301, published online 16 May 2017

In this article the article number was incorrectly given as 15310 but should have been 15301. The original article HTML has now been corrected. The PDF is unaffected.

