# Supplementary material for: Power generator driven by Maxwell's demon
Source: Nat Commun. 2017 May 16;8:15310. doi: 10.1038/ncomms15301 (PMC5440804; doi:10.1038/ncomms15301)
Supplement: Supplementary Information — Supplementary Figures, Supplementary Notes and Supplementary References [file ncomms15301-s1.pdf]

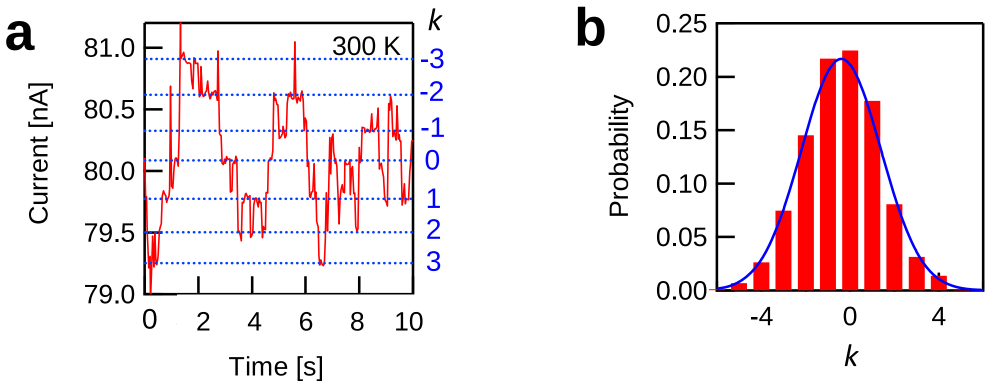


Supplementary Figure 1. **Single-electron detection using a charge sensor.** (**a**) Single-electron detection at room temperature. Current flowing through the sensor is monitored and its abrupt reduction with the same step height means a change in the number of electrons, *n*, in the single-electron box (SEB). The *k* is the deviation from the average of *n*. (**b**) Histogram of *k* when G1 and G2 are open and closed, respectively. We applied $V_{ED}$ = 1.00 V, $V_S$ = $V_D$ = -0.60 V, $V_{UG}$ = 3.05 V, $V_{G1}$ = -2.40 V, and $V_{G2}$ = -1.95 V. At these voltages, the transition rate for electrons in the drain to enter the SEB is two orders of magnitude smaller than that for electrons in the source (S). Therefore, electron transition between the S and SEB dominates the electron motion. Since G1 is open and G2 is closed, electrons shuttle mainly between the S and SEB.

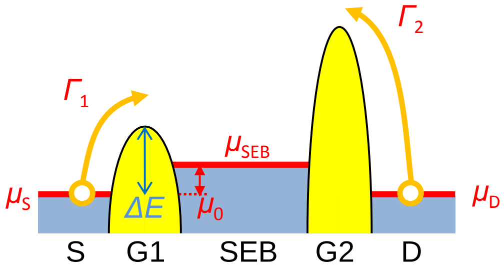


Supplementary Figure 2. **Energy-band diagram along the source (S) and drain (D).** $\mu_S$, $\mu_D$, and $\mu_{SEB}$ are chemical potentials at the S, D, and single-electron box (SEB), respectively. $\Gamma_1$ and $\Gamma_2$ are transition rates from the S to SEB and from the D to SEB, respectively. $\Delta E$ is energy barrier height. $\mu_0$ is the difference in chemical potential between $\mu_S$ and $\mu_{SEB}$.

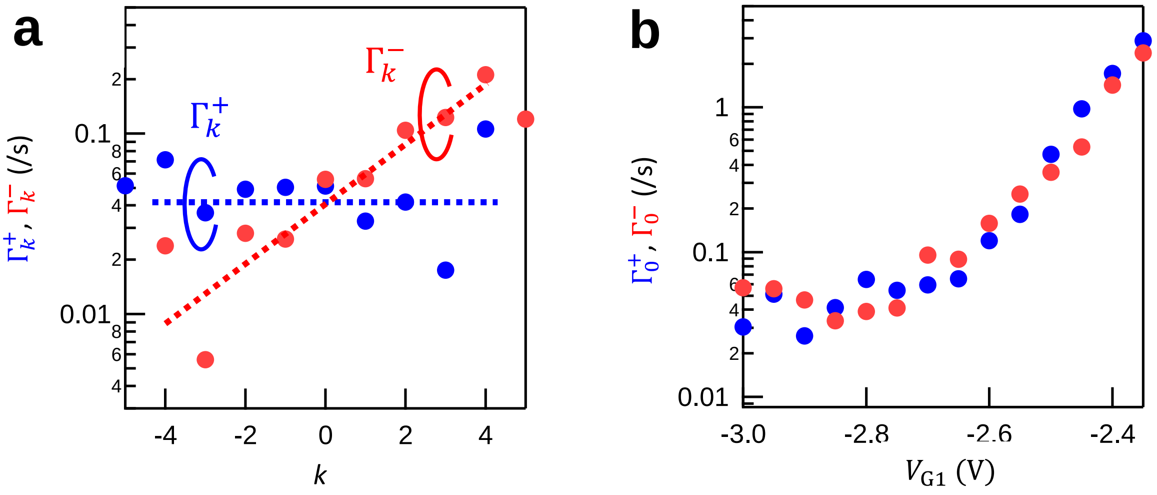


Supplementary Figure 3. **Control of transition rates.** (**a**) Change in transition rates $\Gamma_k^+$ and $\Gamma_k^-$ as a function of *k*. We applied $V_{ED}$ = 0.5 V, $V_S$ = $V_D$ = -0.60 V, $V_{UG}$ = 3.25 V, $V_{G1}$ = -2.95 V, and $V_{G2}$ = -1.85 V. The solid lines are guides for eyes. (**b**) Change in transition rate Γ at *k*=0 as a function of G1 voltage. We applied $V_{ED}$ = 0.5 V, $V_S$ = $V_D$ = -0.60 V, $V_{UG}$ = 3.25 V, and $V_{G2}$ = -1.85 V.

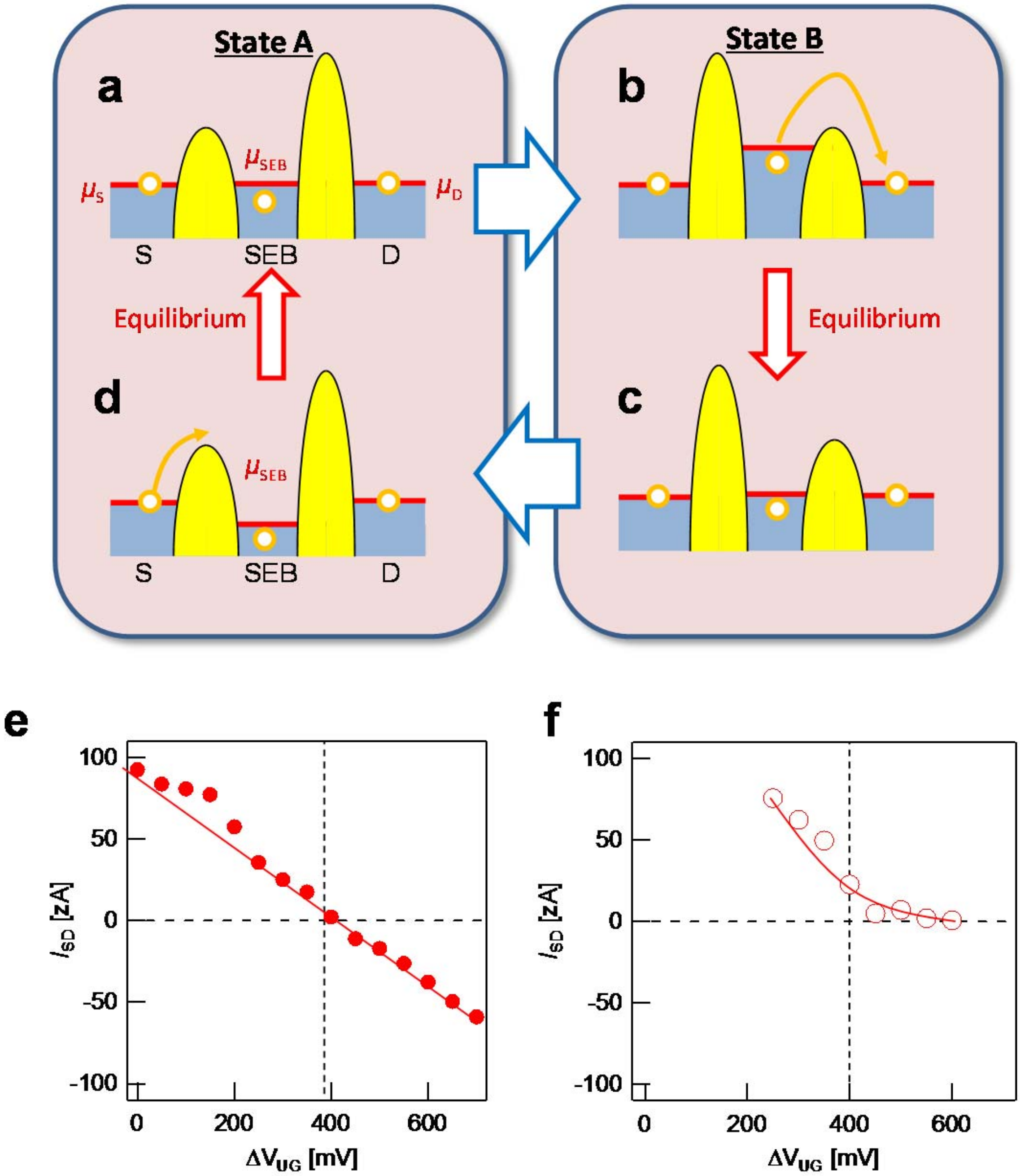


Supplementary Figure 4. **Electron ratchet mechanism: electron transport generated by the change in $\mu_{SEB}$.** (**a-d**) Schematics of electron transport without feedback. (**e, f**) Current flowing from the source (S) and drain (D) as a function of the upper-gate-voltage difference $\Delta V_{UG}$ between state A and B, without and with the feedback process, respectively. The solid lines are guides for the eyes. We applied $V_{ED}$ = 1 V, $V_S$ = $V_D$ = -0.6 V for the states; $V_{UG}$ = 2.7 + $\Delta V_{UG}$ V, $V_{G1}$ = -2.4 V, and $V_{G2}$ = -1.95 V for state A; $V_{UG}$ = 2.7 V, $V_{G1}$ = -3 V, and $V_{G2}$ = -1.6 V for state B.

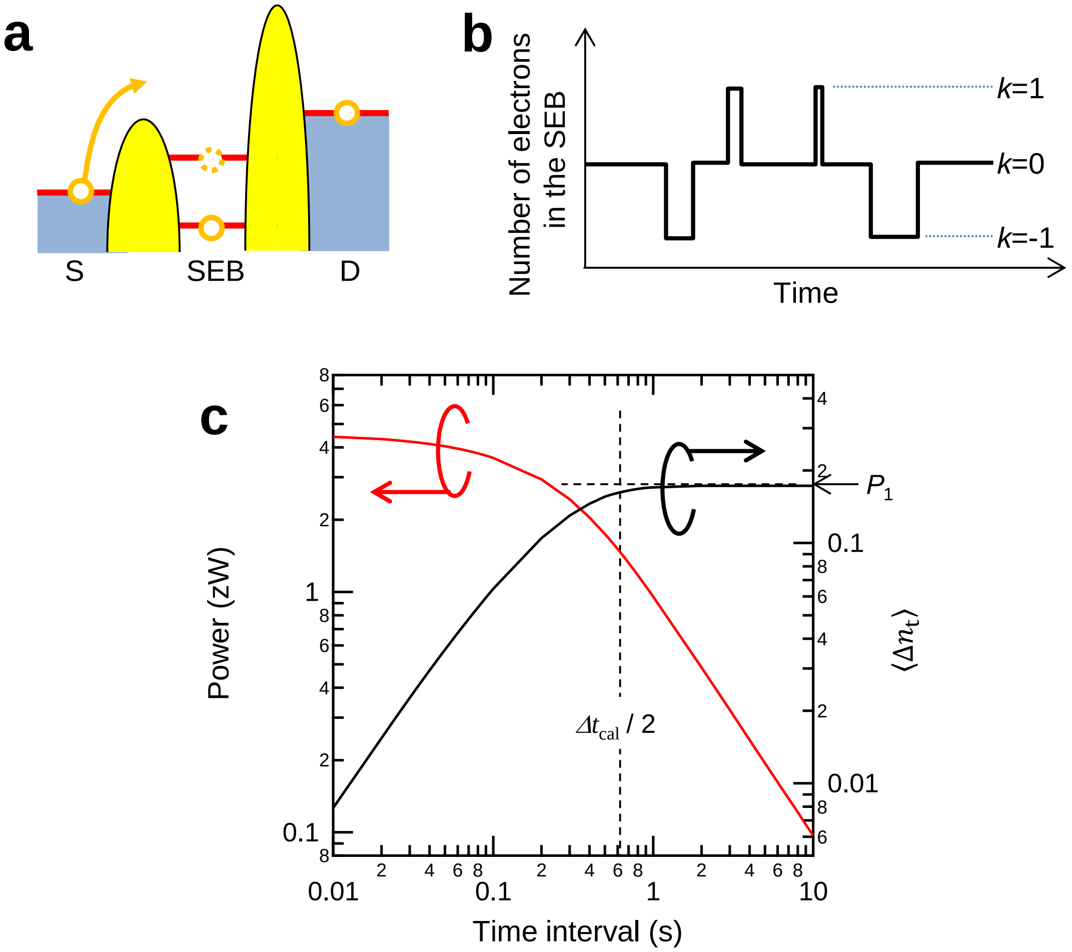


Supplementary Figure 5. **Estimation of optimal measurement interval.** (**a,b**) Schematics of electron shuttling between the single-electron box and source. We assume that $E_C$ = 35 meV, $\Gamma_1$ = 1 s$^{-1}$, $\Gamma_2$ = 0 s$^{-1}$, and $V_D = -2E_C/e$. (c) Simulated ensemble averaged number of transferred electrons,$\langle \Delta n_t \rangle$, per one measurement cycle $\Delta t_m$. The simulation was carried out with Monte-Carlo method. $\Delta t_{cal}$ (=1.26 s) is the average time interval for $k$ of 0 to become 1, evaluated from the numerical calculation, as explained in Supplementary Section 2. $P_1$(=0.171) is the probability that $k$ = 1 in the Gaussian distribution given by (11) and (12).

**Supplementary Note 1: Measurement system and feedback control**

The number of electrons, $n$, in the SEB is monitored in real time with a charge sensor capacitively coupled to the SEB[1] as shown in Supplementary Fig. 1a. The time interval for each measurement $\Delta t_{\mathrm{m}}$ is 60 ms for both states, A and B. For the feedback, when $n$ increases (decreases) from the initially observed $n$ at state A (B), voltages applied to G1 and G2 change. This feedback takes about 20 ms. All measurements were carried out at room temperature and performed with an Agilent B1500.

In our experiments, we consider the deviation $k$ of $n$ from the average of $n$. Supplementary Fig. 1b shows a histogram of $k$ when G1 opens and G2 closes. It shows a Gaussian distribution, which means that electrons shuttle randomly between the source and SEB due to thermal agitation. From the variance $\sigma^2$ of this distribution, we can evaluate the charging energy $E_{\mathrm{C}} = e^2/2C = k_{\mathrm{B}}T/2\sigma^2$, where $e$ is the elementary charge, $C$ is the capacitance of the SEB, $k_{\mathrm{B}}$ is Boltzmann's constant, and $T$ is temperature.

It should be noted that there is large parasitic energy consumption in the present device and in the measurement and feedback systems. Such consumption could be significantly reduced by integrating a feedback controller on a chip or making the demon autonomous. Furthermore, an essential tradeoff between the power consumption and precision for the demon's measurements[2] has recently been pointed out. It will be interesting in future work to study the optimal measurement precision for realizing an efficient power generator driven by Maxwell's demon.

**Supplementary Note 2: Monte-Carlo simulation and numerical calculation for evaluating $I_{\mathbf{M}D}$**

The number of electrons, $n$, in the SEB fluctuates due to thermal energy. For simplicity, we consider the deviation $k$ of $n$ from its average $N_{\mathrm{aver}}$, $k = n - N_{\mathrm{aver}}$.

Transition rates $\Gamma_k^+$ and $\Gamma_k^-$ for $k$ to increase and decrease by one, respectively, are given by

$$\Gamma_k^+ = \Gamma_1 + \Gamma_2 \exp\left(\frac{-V_{\mathrm{D}}}{k_{\mathrm{B}}T}\right), \text{and} \tag{1}$$

$$\Gamma_k^- = \Gamma_1 \exp\left(\frac{\mu_0 + 2(k-0.5)E_{\mathrm{C}}}{k_{\mathrm{B}}T}\right) + \Gamma_2 \exp\left(\frac{\mu_0 + 2(k-0.5)E_{\mathrm{C}}}{k_{\mathrm{B}}T}\right), \tag{2}$$

where $\Gamma_1$ is the transition rate for an electron to enter from the source to the SEB, $\Gamma_2$ is the transition rate for an electron to enter from the drain to the SEB at $V_{\mathrm{SD}} = 0$ V, $V_{\mathrm{SD}}$

is source-drain bias voltage, $k_{\mathrm{B}}$ is Boltzmann's constant, $T$ is temperature, and $\mu_0$ is the difference in chemical potential between the source and SEB when $k = 0$, and $E_{\mathrm{C}} = e^2/2C$ (see Supplementary Fig. 2). The first and second parts in both (1) and (2) depict rates for electrons to surmount energy barriers formed by G1 and G2, respectively.

In Monte-Carlo simulation for Figs. 3, 4a, and 4c, we used $\mu_0 = 0$ meV, $E_{\mathrm{C}} = 4.2$ meV, $\Gamma_1 = 1.2$ and $\Gamma_2 = 0.01$ at state A, $\Gamma_1 = 0.01$ and $\Gamma_2 = 1.4$ at state B, all of which are close to the experimentally obtained values. In the experiments, time intervals for each measurement and feedback were 60 and 20 ms, respectively. In order to deal with these time intervals in the simulation, transient characteristics of the $n$ in the SEB were first simulated and then $n$ at the timings of each measurement and feedback was obtained. Although Monte-Carlo simulation enables us to consider time intervals for each measurement and feedback easily, there is a tradeoff between speed and accuracy. To guarantee high speed and high accuracy in estimating fundamental performance regardless of experimental conditions, such as the time intervals mentioned above, we used numerical simulation to estimate the ideal performance without the time intervals.

For the numerical simulations, we have to consider other parameters. From (1) and (2), we can evaluate probabilities $P_k^+$ and $P_k^-$ for $k$ to increase and decrease by one, respectively, and the time interval $\tau_k$ until the change in $k$ as follows:

$$P_k^{+(-)} = \frac{\Gamma_k^{+(-)}}{\Gamma_k^+ + \Gamma_k^-} \text{ and} \tag{3}$$

$$\tau_k = \frac{1}{\Gamma_k^+ + \Gamma_k^-}. \tag{4}$$

Using these equations, we can evaluate the time interval $T_k$ for $k$ to become $\Delta n$ ($> k$) at state A with consideration of the idea of a random walk:

$$T_k = \tau_k + P_k^- T_{k-1} \ (k = \Delta n - 1), \tag{5}$$

$$T_k = \tau_k + P_k^- T_{k-1} + P_k^+ T_{k+1} \ (k < \Delta n - 1). \tag{6}$$

Time interval $T_k'$ for $k'$ to become 0 at state B is given by

$$T_{k'}' = \tau_{k'} + P_{k'}^- T_{k'-1}' \ (k' = 1), \text{ and} \tag{7}$$

$$T_{k'}' = \tau_{k'} + P_{k'}^- T_{k'-1}' + P_{k'}^+ T_{k'+1}' \ (k' > 1). \tag{8}$$

For the calculation, we have to define the boundary of $k$. The range of $k$ corresponds to that of the thermal fluctuation of $n$, and its standard deviation $\sigma$ is given by $(k_{\mathrm{B}}T/2E_{\mathrm{C}})^{0.5}$,

where $E_\mathrm{C} = e^2/2C$ is the charging energy. In our experiments, $E_\mathrm{C} = 4$ meV and $\sigma = 1.8$. Therefore, assuming the probability of $|k| > 30$ corresponding to $17\sigma$ is almost zero, we evaluate $T_k$ and $T'_k$ from the equations in the range of $|k| \leq 30$.

From (1)–(8), we evaluate current $I = e/(T_k + T'_k)$. However, since the direction of electron motion is not considered in this calculation, we need to isolate ideal current $I_\mathrm{MD}$ flowing from the drain to source from $I$ evaluated above:

$$I_\mathrm{MD} = \frac{e}{T_k + T'_k} \frac{\Gamma_{1,\mathrm{A}}}{\Gamma_{1,\mathrm{A}} + \Gamma_{2,\mathrm{A}} \exp\left(-V_\mathrm{D}/k_\mathrm{B}T\right)} \frac{\Gamma_{1,\mathrm{B}}}{\Gamma_{1,\mathrm{B}} + \Gamma_{2,\mathrm{B}}}, \tag{9}$$

where $\Gamma_{1,\mathrm{A}}$ and $\Gamma_{1,\mathrm{B}}$ are the transition rates for an electron in the source to enter the SEB at state A and B, respectively, and $\Gamma_{2,\mathrm{A}}$ and $\Gamma_{2,\mathrm{B}}$ are the transition rates for an electron in the drain to enter the SEB at state A and B, respectively. In Fig. 4d,e, we evaluate the power with numerically calculated $I_\mathrm{MD}$. Note that the calculated $I_\mathrm{MD}$ corresponds to the condition with $\Delta t_\mathrm{m} = 0$ and gives a rough estimation for the conditions with $\Delta t_\mathrm{m}$ comparable to $T_k$. To estimate an exact value when $\Delta t_\mathrm{m}$ is non-zero, we need to perform a Monte-Carlo simulation using $\Gamma_k^+$ and $\Gamma_k^-$.

**Supplementary Note 3: Experimental evaluation of transition rate**

Since there are two energy barriers formed by G1 and G2, the transition rates for electrons to enter the SEB through each energy barrier must be estimated separately. When G1 is open and G2 is closed, the probability that an electron in the drain enters the SEB is much smaller than that for an electron in the source. In this case, the transition rate $\Gamma_{1,k}^\pm$ for an electron to enter (+) and leave (-) the SEB through the energy barrier formed by G1 when $n = k + N_\mathrm{aver}$ is estimated to be

$$\Gamma_{1,k}^\pm = \frac{1}{\langle \tau_k \rangle} \frac{P_k^\pm}{P_k^+ + P_k^-}. \tag{10}$$

Supplementary Fig. 3a shows $\Gamma_{1,k}^\pm$ as a function of $k$. The dependence of $\Gamma_{1,k}^+$ on $k$ is weaker than that of $\Gamma_{1,k}^-$. The reason for the weak dependence of $\Gamma_{1,k}^+$ on $k$ is that the difference between the top of the energy barrier and chemical potential of the source is constant and independent of $k$, which corresponds to the first part of the right side of (1). On the other hand, in the case of $\Gamma_{1,k}^-$, the difference between the top of the energy barrier and chemical potential of the SEB changes with $k$ , which corresponds to the first part of the right side of (2).

Supplementary Fig. 3b shows $\Gamma^{+}_{1,0}$ as a function of voltage $V_{\mathrm{G1}}$ applied to G1. $\Gamma^{+}_{1,0}$ corresponds ideally to $\Gamma_1$ in (1). $\Gamma^{+}_{1,0}$ changes exponentially with $V_{\mathrm{G1}}$ because an increase in $V_{\mathrm{G1}}$ reduces the height of the energy barrier formed by G1.

In the same way, by closing G1 and opening G2, we estimate the transition rate for an electron to shuttle between the SEB and drain through the energy barrier formed by G2.

It is impossible for the energy barrier to stop an electron from shuttling at finite temperature, which means that there is undesirable electron shuttling between, for instance, the SEB and drain at state A. However, we can separate the desirable and undesirable electron shuttling mathematically by using the experimentally obtained transition rates for electrons to surmount energy barriers formed by G1 and G2. In our experiment, the desirable shuttling dominates about 98% of the measured results.

## Supplementary Note 4: Current generation based on mechanism of single-electron ratchet

One of the main topics of this paper is current generation by the feedback process performed by Maxwell's demon. Another way to generate current using two energy barriers formed by transistors is to use a single-electron ratchet mechanism. This mechanism is available when the chemical potential of the SEB is modulated by the gates of transistors due to the capacitive coupling between the SEB and gates.

For a simple explanation, we consider the case at state A as shown in Supplementary Fig. 4a. At equilibrium, the chemical potential $\mu_{\mathrm{S}}$, $\mu_{\mathrm{D}}$, and $\mu_{\mathrm{SEB}}$ of the source, drain, and SEB, respectively, are aligned with each other. When the state switches from A to B and gate voltages applied to G1 and G2 are changed, $\mu_{\mathrm{SEB}}$ is also changed because capacitive coupling between the SEB and G1 is different from that between the SEB and G2. For example, when $\mu_{\mathrm{SEB}}$ rises as shown in Supplementary Fig. 4b, electrons in the SEB escape from the SEB to the drain and then, finally, $\mu_{\mathrm{S}}$, $\mu_{\mathrm{D}}$, and $\mu_{\mathrm{SEB}}$ are aligned again at equilibrium (Supplementary Fig. 4c). Next, the state switches from B to A, and $\mu_{\mathrm{SEB}}$ drops (Supplementary Fig. 4d) due to capacitive coupling and electron removal as shown in Supplementary Fig. 4b. In order to put the system in equilibrium as shown in Supplementary Fig. 4a, electrons in the source enter the SEB. Sequentially, this cycle transfers electrons in the source to the SEB and then to the drain, even without feedback. On the other hand, when the state is switched from A to B and then $\mu_{\mathrm{SEB}}$ drops, electrons are transferred from the drain to source by the

same cycle.

In order to suppress modulation in $\mu_{\mathrm{SEB}}$ due to the capacitive coupling, we used another gate which covers the whole area. Since this gate, hereafter referred to as upper gate UG, is capacitively coupled to the SEB, it can control $\mu_{\mathrm{SEB}}$ individually and then suppress the change in $\mu_{\mathrm{SEB}}$ when the condition is changed between state A and B.

Supplementary Fig. 4e shows current as a function of the UG-voltage difference $\Delta V_{\mathrm{UG}}$ between state A and B, i.e., $\Delta V_{\mathrm{UG}} = V_{\mathrm{UG,B}} - V_{\mathrm{UG,A}}$, where $V_{\mathrm{UG,A(B)}}$ is UG voltage at state A (B), without the feedback process. Current decreases linearly with increasing $\Delta V_{\mathrm{UG}}$. This reduction occurs because at the step shown by Supplementary Figs. 4b and d, $\Delta V_{\mathrm{UG}}$ suppresses the change in $\mu_{\mathrm{SEB}}$ and suppresses electron removal from it and injection to the SEB, respectively. At $\Delta V_{\mathrm{UG}} = 0.4$ V, current becomes zero, which indicates there is no $\mu_{\mathrm{SEB}}$ modulation at the SEB. $\Delta V_{\mathrm{UG}}$ larger than 0.4 V decreases $\mu_{\mathrm{SEB}}$, compared to the step shown in Supplementary Fig. 4a, and thus electrons in the drain enter the SEB, which leads to electron transfer from the drain to source. With these experiments, we eliminate current generated by the single-electron ratchet mechanism and monitor current generated by Maxwell's demon at $\Delta V_{\mathrm{UG}} = 0.4$ V. Otherwise, at $\Delta V_{\mathrm{UG}} \neq 0.4$ V, current is generated with the single-electron ratchet mechanism (Supplementary Fig. 4f).

The current $I_{\mathrm{SD}}$ passing through the SEB is estimated from ensemble average of the number of transferred electrons, $\langle \Delta n_{\mathrm{t}} \rangle$. To equilibrate $n$, we wait 4 s before switching between state A and B. Therefore, one cycle consumes $\Delta t_{\mathrm{cyc}} = 8$ s to transfer electrons from the source to drain. The number of transferred electrons, $\Delta n_{\mathrm{t}}$, in a cycle is defined as $\Delta n_{\mathrm{t}} = (\Delta n_{\mathrm{A}} + \Delta n_{\mathrm{B}})/2$, where $\Delta n_{\mathrm{A}}$ is the increase in $n$ in state A and $\Delta n_{\mathrm{B}}$ is the decrease in $n$ in state B. By the definition of the current, $I_{\mathrm{SD}}$ is given by $\langle \Delta n_{\mathrm{t}} \rangle e/\Delta t_{\mathrm{cyc}}$.

We estimate the modulation $\Delta\mu_{\mathrm{SEB}}$ in the chemical potential at the SEB caused by the switching between the state A and B using $I_{\mathrm{SD}}$ as follows. Finite $I_{\mathrm{SD}}$ means that $\langle \Delta n_{\mathrm{t}} \rangle$ and $\langle \Delta\mu_{\mathrm{SEB}} \rangle$ are not zero. Since the SEB has charging energy $E_{\mathrm{C}} = e^2/2C = 4$ mV, an increase of a single electron in the SEB results in an increase in $\mu_{\mathrm{SEB}}$ by $e^2/C$, and $\Delta\mu_{\mathrm{SEB}}$ is given by $\Delta n_{\mathrm{t}} \times 2E_{\mathrm{C}} = I_{\mathrm{SD}} \times (\Delta t_{\mathrm{cyc}}/e) \times 2E_{\mathrm{C}}$.

## Supplementary Note 5: Protocol for improving efficiency

We can perform a simulation using the Monte-Carlo method to find an optimized condition to improve the efficiency considering the tradeoff between efficiency and power. For simplicity,

we consider the case that at state A, $E_C$ (=35 meV) is large enough so that just one electron shuttles between the SEB and source, i.e., $k$ = -1, 0, or 1, as shown in Supplementary Figs. 5a and b. Now, we consider the dependence of the ensemble averaged number $\langle \Delta n_t \rangle$ of transferred electrons per one measurement on time interval $\Delta t_m$. Supplementary Fig. 5c shows $\langle \Delta n_t \rangle$ as a function of $\Delta t_m$. $\Delta n_t$ increases as $\Delta t_m$ increases until $\Delta t_m$ exceeds $\Delta t_{cal}$, which is the time interval for $k$ of 0 to become 1, evaluated from the numerical calculation described above, and finally reaches $P_1$ with $\Delta t_m$ larger than $\Delta t_{cal}$. This behavior can be understood from the sampling theory based on the Nyquist frequency. When $\Delta t_m$ is shorter than $\Delta t_{cal}/2$, the transient characteristics can be measured with statistically high precision as shown in Supplementary Fig. 5b. However, for most of the measurements, $k$ is still zero and no feedback is applied, which reduces the efficiency. On the other hand, when $\Delta t_m$ is longer than $\Delta t_{cal}/2$, the transient characteristics cannot be measured precisely. Instead, $k$ of 1 is measured with the probability based on a Gaussian distribution: The probability $P_i$ that $k = i$ is given by

$$P_i = P_0 \exp\left(-\frac{i^2 E_C}{k_B T}\right) \text{ and} \tag{11}$$

$$P_0 = \sum_{i=-\infty}^{\infty} \exp\left(-\frac{i^2 E_C}{k_B T}\right). \tag{12}$$

Therefore, the increase in $\Delta t_m$ makes $\langle \Delta n_t \rangle$ become $P_1$ as shown in Supplementary Fig. 5c. Subsequently, in order to improve the efficiency for converting information to energy, $\Delta t_m$ should be adjusted so that it becomes longer than $\Delta t_{cal}/2$. However, since $\langle \Delta n_t \rangle$ saturates when $\Delta t_m$ is larger than $\Delta t_{cal}/2$, longer $\Delta t_m$ increases the time interval for Maxwell's demon to detect that $k$ becomes $\Delta n$ as shown in Supplementary Fig. 5c and thus reduces the current generated by the demon. So, $\Delta t_m \sim \Delta t_{cal}/2$ provide us better efficiency and larger current. As easily expected, this idea is adaptable to the case of state B. In this case, $\Delta t_m$ must be changed according to the time interval for $k$ to become zero.

**Supplementary Note 6: Effect of detection errors**

The detection errors decrease the amount of information usable for Maxwell's demon[3]. We obtain measurement result $m$ when the number of electrons in the SEB is $n$. We define $N$ and $M$ as 0 when $n < \Delta n_{thresh}$ and $m < \Delta n_{thresh}$, respectively, and they are 1 otherwise. $\Delta n_{thresh}$ is the threshold value for the feedback control. The mutual information $I$ is given by

$\sum_{n,m} P(N,M)I(N,M)$, where $P(N,M)$ is the joint probability of $N$ and $M$ and $I(N,M) = H_N(N) + H_M(M) - H(N,M)$, where $H_N(N)$ is the Shannon entropy of $N$, $H_M(M)$ is that of $M$, and $H(N,M)$ is the joint Shannon entropy of $N$ and $M$. With the error rate of the measurement $\varepsilon$, we obtain the probability of $N$ as $P_N(0) = p$ and $P_N(1) = 1-p$; the probability of $M$ as $P_M(0) = p(1-\varepsilon) + (1-p)\varepsilon$ and $P_M(1) = p\varepsilon + (1-p)(1-\varepsilon)$; $P(0,0) = p(1-\varepsilon)$, $P(0,1) = p\varepsilon$, $P(1,0) = (1-p)\varepsilon$, and $P(1,1) = (1-p)(1-\varepsilon)$. As a result, $I$ is given by $-p(1-\varepsilon)\ln\left(p + \frac{(1-p)\varepsilon}{1-\varepsilon}\right) - p\varepsilon \ln\left(p + \frac{(1-p)(1-\varepsilon)}{\varepsilon}\right) - (1-p)\varepsilon \ln\left(\frac{p(1-\varepsilon)}{\varepsilon} + (1-p)\right) - (1-p)(1-\varepsilon)\ln\left(\frac{p\varepsilon}{1-\varepsilon} + (1-p)\right)$. In particular, when $p = 1/2$, $I$ becomes $\ln 2 + (1-\varepsilon)\ln(1-\varepsilon) + \varepsilon \ln \varepsilon$. With error-free measurements ($\varepsilon = 1$) or all-false measurements ($\varepsilon = 0$), $I$ equals to $H_N$. By increasing (decreasing) $\varepsilon$ from 0 (1), $I$ decreases until $\varepsilon$ becomes 0.5. With $\varepsilon = 0.5$, $I$ becomes zero.

$I$ represents the amount of available information. $I$ become maximum with error-free measurements and an increase in $\varepsilon(< 0.5)$ decreases it. Our definition of the efficiency of the information-to-energy conversion $F/k_{\mathrm{B}}TI$ is the ratio between the generated free energy and the available information. Therefore, the efficiency becomes large when the available information is decreased by considering $\varepsilon(< 0.5)$.

In our measurements, $\varepsilon$ was about 0.08. This increases the efficiency at the maximum power in the experiments and the Monte-Carlo simulation to $\sim 30\%$ and 40%, respectively. However, since we did not evaluate $\varepsilon$ experimentally[3], we evaluated the efficiency by assuming $I = H_N$ to obtain its lower bound.

## SUPPLEMENTARY REFERENCES

[1] Nishiguchi, K. *et al.* Single-electron-resolution electrometer based on field-effect transistor. *Jpn. J. Appl. Phys.* **47**, 8305 (2008).

[2] Barato, A. C., & Seifert, U. Thermodynamic uncertainty relation for biomolecular processes. *Phys. Rev. Lett.* **114**, 158101 (2015).

[3] Koski, J., Maisi, V., Sagawa, T. & Pekola, J. Experimental Observation of the Role of Mutual Information in the Nonequilibrium Dynamics of a Maxwell Demon. *Phys. Rev. Lett.* **113**, 030601 (2014).
